# Correlated Activity in the Degenerate Retina Inhibits Focal Response to Electrical Stimulation

**DOI:** 10.3389/fncel.2022.889663

**Published:** 2022-05-04

**Authors:** Jungryul Ahn, Seongkwang Cha, Kwang-Eon Choi, Seong-Woo Kim, Yongseok Yoo, Yong Sook Goo

**Affiliations:** ^1^Department of Physiology, Chungbuk National University School of Medicine, Cheongju, South Korea; ^2^Department of Ophthalmology, Korea University College of Medicine, Seoul, South Korea; ^3^Department of Electronics Engineering, Incheon National University, Incheon, South Korea

**Keywords:** retinal degeneration, electrical stimulation, *rd1* mice, *rd10* mice, non-human primate model, retinal ganglion cell, retinal network, correlation analysis

## Abstract

Retinal prostheses have shown some clinical success in patients with retinitis pigmentosa and age-related macular degeneration. However, even after the implantation of a retinal prosthesis, the patient’s visual acuity is at best less than 20/420. Reduced visual acuity may be explained by a decrease in the signal-to-noise ratio due to the spontaneous hyperactivity of retinal ganglion cells (RGCs) found in degenerate retinas. Unfortunately, abnormal retinal rewiring, commonly observed in degenerate retinas, has rarely been considered for the development of retinal prostheses. The purpose of this study was to investigate the aberrant retinal network response to electrical stimulation in terms of the spatial distribution of the electrically evoked RGC population. An 8 × 8 multielectrode array was used to measure the spiking activity of the RGC population. RGC spikes were recorded in wild-type [C57BL/6J; P56 (postnatal day 56)], *rd1* (P56), *rd10* (P14 and P56) mice, and macaque [wild-type and drug-induced retinal degeneration (RD) model] retinas. First, we performed a spike correlation analysis between RGCs to determine RGC connectivity. No correlation was observed between RGCs in the control group, including wild-type mice, *rd10* P14 mice, and wild-type macaque retinas. In contrast, for the RD group, including *rd1, rd10* P56, and RD macaque retinas, RGCs, up to approximately 400–600 μm apart, were significantly correlated. Moreover, to investigate the RGC population response to electrical stimulation, the number of electrically evoked RGC spikes was measured as a function of the distance between the stimulation and recording electrodes. With an increase in the interelectrode distance, the number of electrically evoked RGC spikes decreased exponentially in the control group. In contrast, electrically evoked RGC spikes were observed throughout the retina in the RD group, regardless of the inter-electrode distance. Taken together, in the degenerate retina, a more strongly coupled retinal network resulted in the widespread distribution of electrically evoked RGC spikes. This finding could explain the low-resolution vision in prosthesis-implanted patients.

## Introduction

Retinal prostheses aim to restore vision in the blind with retinal degeneration, such as retinitis pigmentosa (RP) and age-related macular degeneration (Weiland et al., 2011; Farvardin et al., 2018; Bloch et al., 2019; Palanker et al., 2020). The strategy of the retinal prosthesis is to activate intact retinal neurons, including bipolar cells or retinal ganglion cells (RGCs), bypassing degenerate photoreceptors.

Although retinal prostheses have shown some clinical success, a patient’s visual acuity restored with electronic retinal devices is at best less than 20/420 (Stingl et al., 2013; Ayton et al., 2020). To improve visual acuity, many engineers have attempted to increase the spatial resolution of electrical devices by integrating a large number of smaller electrodes on an electronic chip (Zeng et al., 2019; Shire et al., 2020; Chenais et al., 2021b), optimizing electrode configurations (Wilke et al., 2011; Celik, 2017; Flores et al., 2018) or stacking more electrodes in a three-dimensional structure (Bendali et al., 2015; Davidsen et al., 2019; Seo et al., 2019). Physiologists have searched for optimal stimulation protocols, such as the minimum stimulation threshold required for local activation of target neurons to avoid axon bundle activation or non-specific activation of nearby RGCs (Sekirnjak et al., 2008; Jepson et al., 2013; Grosberg et al., 2017; Chang et al., 2019; Tandon et al., 2021).

As another physiological strategy for improving vision, the activation of surviving photoreceptors or bipolar cells has emerged as a novel approach that mimics the natural visual processing of the normal retina. Electrical activation of retinal neurons upstream of RGCs shows a burst-like physiological response similar to a light response, whereas direct RGC activation exhibits only a single spike. Thus, we call this response a network-mediated response originating from upstream neurons, such as bipolar cells and photoreceptors (Boinagrov et al., 2014; Im and Fried, 2015; Haq et al., 2018; Im et al., 2018).

Degenerate retina shows retinal remodeling and abnormal neural connections in the retinal network (Marc et al., 2003; Menzler and Zeck, 2011; Margolis et al., 2014; Jones et al., 2016). During photoreceptor degeneration, a significant rewiring process converts the retina into self-signaling neural networks, resulting in spontaneous hyperactivity. A possible physiological explanation of the low vision found in RP patients is a decrease in the signal-to-noise ratio (SNR) due to spontaneous hyperactivity of the RGCs (Goo et al., 2011b,^2016^; Euler and Schubert, 2015; Ivanova et al., 2016; Haselier et al., 2017). This induces a less reliable RGC response to repetitive electrical stimulation (Ho et al., 2020; Yoon et al., 2020). Therefore, higher stimulation intensities may be required to electrically activate degenerate RGCs than that required for normal RGCs (Jensen and Rizzo, 2009; Goo et al., 2011b; Cha et al., 2021). However, all previous experiments have focused on the single RGC level response, not the population of RGC responses.

Therefore, in this study, we focused on the spatial distribution of electrically evoked RGC population responses. Through pharmacological experiments, we identified the neuronal mechanism underlying the aberrant spatial distribution of RGC population responses in the degenerate retina. This may be considered a retinal origin of low-resolution vision even after retinal prosthesis implantation. Therefore, correction of abnormal rewiring could enable better visual acuity with retinal prostheses in the future.

## Materials and Methods

### Animals

We used four types of mice: C57BL/6J strain (wild-type) at postnatal day 56 (P56) (*n* = 14), C3H/HeJ (*rd1*) at P56 (*n* = 25), B6CXB1-*Pde6b^rd10^*/J (*rd10*) at P14 (*n* = 5), and *rd10* at P56 (*n* = 6). In this study, we compared the control and retinal degeneration (RD) mouse groups: wild-type (P56) *vs. rd1* (P56), and *rd10* (P14) *vs. rd10* (P56). At P56 in *rd1* mice, the retinas are no longer responsive to light, but the functional stability of the inner retinal neurons is well preserved (Margolis et al., 2008). At P14 in *rd10* mice, photoreceptors are almost conserved, similar to the wild-type (Chang et al., 2007; Gargini et al., 2007; Stasheff et al., 2011). In contrast, at P56 in *rd10* mice, retinal light responses were almost lost, but the functional stability of inner retinal neurons was well preserved. All mice were purchased from Jackson Laboratories (The Jackson Laboratory, Bar Harbor, ME, United States). Animal use protocols were approved by the Institutional Animal Care Committee of Chungbuk National University (approval number: CBNUA-1172-18-02). All the procedures followed the guidelines of the Association for Research in Vision and Ophthalmology Statement for the Use of Animals in Ophthalmic and Vision Research.

All *in vitro* macaque experiments were performed in accordance with ARRIVE guidelines [Institutional Animal Care Committee of the OSONG KBIO HEALTH (approval number: KBIO-IACUC-2020-054)]. We used three cynomolgus monkeys (*Macaca fascicularis*) as wild-type controls and two cynomolgus monkeys as the RD model induced by *N*-methyl-*N*-nitrosourea (MNU). Their average age was 49.8 ± 2.8 months, and their average body weight was 3.87 ± 0.65 kg. The detailed procedures for developing the RD macaque model are described in a previous study (Kim, 2021; Choi, 2022). Briefly, the RD model was induced in the right eye by intravitreal loading of MNU (concentration: >2 mg/mL, exposure time: 10 min), which selectively induces photoreceptor apoptosis. Supplementary Figures 1, 2 showed the anatomical and electrophysiological retinal changes in the RD model 12 weeks after intravitreal MNU injection using *in vivo* assessments based on optical coherence tomography (OCT) and electroretinography (ERG). There was no significant change in retinal thickness before and after MNU injection in the foveal region with high cone cell density. However, in the periphery where the rod cell density is relatively high, the retinal thickness decreases from the retinal pigment epithelium (RPE) to the outer nuclear layer (ONL). Thus, the MNU-induced RD macaque model mimics RP in patients with peripheral visual field defects. For further *in vitro* macaque experiments, degenerate peripheral retinal regions were used. The subjects were sacrificed approximately 3 months after MNU administration.

### Retinal Preparation

Mice were anesthetized by intramuscular injection of 30 mg/kg tiletamine-zolazepam hydroxide (Zoletil 50; Virbac, São Paulo, Brazil), 10 mg/kg zylazine hydrochloride (Rumpun; Bayer Korea, Seoul, South Korea), and 5,000 IU heparin sodium (Heparin; JW Pharmaceutical Corp., Seoul, South Korea). Macaque monkeys were anesthetized with an intravenous injection of 1 mg/kg alfaxalone (Alfaxan; Vetoquinol United Kingdom, Towcester, United Kingdom) into the marginal auricular vein following premedication, which comprised a subcutaneous injection of 0.05 mg/kg atropine, intramuscular injection of 1 mg/kg xylazine (Rompun; Bayer Corp., Shawnee Mission, KA, United States), and 4 mg/kg azaperone (Stresnil; Mallinckrodt Veterinary Inc., Indianapolis, IN, United States). The subjects were euthanized immediately after the enucleation of the eyeball.

The detailed procedures for the preparation of *ex vivo* retinal patches have been described in previous studies (Stett et al., 2000; Ahn et al., 2015). Briefly, after eye enucleation, the retina was isolated from the sclera and RPE and cut into 2 × 2 mm^2^ patches. The retinal patch was prepared under the illumination of 4.3 nW/cm^2^ in an artificial cerebrospinal fluid solution (124 mM NaCl, 10 mM glucose, 1.15 mM KH_2_PO_4_, 25 mM NaHCO_3_, 1.15 mM MgSO_4_, 2.5 mM CaCl_2_, and 5 mM KCl) bubbled with 95% O_2_ and 5% CO_2_ to maintain a pH of 7.3–7.4 and a temperature of 32°C. The isolated retina was mounted on the RGC layer on a planar multi-electrode array (MEA) and continuously perfused with oxygenated solution (flow rate: 1–3 mL/min) during the experiment.

### Multi-Electrode Recording System and Signal Processing

The data acquisition system (MEA60 system; Multichannel Systems GmbH, Reutlingen, Germany) included a planar 64-channel perforated MEA (60pMEA200/30iR), an amplifier (MEA1060), temperature control units (TC01), data acquisition hardware (Mc_Card), and software (Mc_Rack). The MEA contained 64 circular electrodes in an 8 × 8 grid layout with electrode diameters of 30 μm and inter-electrode distances of 200 μm. The electrodes were coated with porous titanium nitride and embedded in a perforated polyimide foil that provided sufficient oxygen and nutrient supply to the retina. Multi-electrode recordings of retinal activity were obtained from 59 out of 64 electrodes, except for one reference electrode and four inactive electrodes with a bandwidth ranging from 1 to 3,000 Hz at a gain of 1,200. The data-sampling rate was 25 kHz for each electrode. From the raw waveform of the retinal recording, RGC spikes were isolated using a 100-Hz high-pass filter. Local field potential (LFP) traces were isolated from low-pass filtering using a 40-Hz cutoff frequency. The threshold for spike detection was set to four times the standard deviation of background noise. The recorded data were processed with spike sorting software (Offline Sorter; Plexon Inc., Dallas, TX, United States) for each MEA channel to separate multiunit activities containing different spike waveforms into individual cell units using principal component analysis (Lewicki, 1998). Therefore, for a pair of RGCs, the interelectrode distance is the distance between the RGCs.

### Cross-Correlation Analysis

The cross-correlation of two spike trains simultaneously recorded between the RGCs can be quantified using a cross-correlogram that displays the amount of synchronized firing between the two cells. First, we binned the spike trains in the RGC pairs to generate a binary number of spikes for each RGC, as a function of time. Next, we used the cross-correlation index (CCI) to quantify the strength of the correlation between the two RGCs. It is defined as the ratio between the probability of synchronized firing (i.e., two cells firing together during a time lag) and the expected probability of a statistically independent firing (Shlens et al., 2006). CCI was calculated using the following equation:


(1) $$C\,C\,I=\log_{2}\frac{P\,\left(A,B\right)}{P\,\left(A\right)\,P\,\left(B\right)}$$

where *A* and *B* denote events where RGC A and RGC B spike, respectively. Two independent spike trains had a CCI value of zero [*P*(*A*)*P*(*B*)=*P*(*A*,*B*)], with higher CCI values indicating higher synchrony.

In Equation 1, the time lag for probability calculation was chosen to be 0.2 s (200 ms), considering the fact that retinal remodeling occurs between inner retinal neurons through the gap junction relay between ON-cone bipolar cells and AII amacrine cells in the degenerate retina (Margolis et al., 2014; Trenholm and Awatramani, 2015). The time lag through indirect connections *via* the relays of inner retinal neurons, including bipolar or amacrine cells, which are upstream neurons of the RGC, is within approximately 200 ms (Brivanlou et al., 1998). The CCIs were calculated and plotted for interelectrode distances ranging from the nearest distance of 200–1,600 μm in steps of 200 μm.

### Electrical Stimulation

Using a stimulus generator (STG 1004, Multichannel Systems GmbH, Reutlingen, Germany), the current pulse train was delivered to the retinal preparation through one of the 60 channels (mostly channel 44 in the middle of the MEA), with the remaining channels serving as recording electrodes. The remaining channels of the MEA were binned into five groups (200–400, 400–600, 600–800, 800–1,000, and 1,000–1,200 μm) based on the distance between the stimulation and recording electrodes of the MEA. The stimulation consisted of symmetrical cathodic phase 1st biphasic pulses. The pulse duration was fixed at 500 μs/phase and pulse amplitudes of 5, 10, 20, 30, 40, and 50 μA/phase were applied. For each pulse amplitude, biphasic current pulses were applied 50 times once per second (1 Hz).

### Pharmacological Treatment

The drug solution was applied *via* perfusion for at least 20 min before recording. A combination of 50 μM 6,7-dinitroquinoxaline-2,3-dione (DNQX, 0189, TOCRIS, Bristol, United Kingdom) and 50 μM DL-2-amino-5-phosphonopentanoic acid sodium salt (DL-AP5, 3693, TOCRIS, Bristol, United Kingdom) was used to block the ionotropic glutamate AMPA/kainate receptors and NMDA receptors. Gap junction coupling was blocked using 100 μM meclofenamic acid (MFA; M4531; Sigma-Aldrich, St. Louis, MO, United States).

### Data Analysis

Data analysis was performed using commercial analysis software (NeuroExplorer™; Plexon Inc., Dallas, TX, United States), commercial statistical software (IBM SPSS Statistics 24; IBM Corp., New York, NY, United States), and custom-made MATLAB (MathWorks, Natick, MA, United States) code.

The average spike frequency for the RGCs was calculated as the total number of spikes divided by the total recording time (3 min before electrical stimulation). Fast Fourier transform (FFT) was performed to detect the principal frequencies of LFPs commonly observed in degenerate retinas (Goo et al., 2011a; Yee et al., 2012; Biswas et al., 2014).

The temporal structure of the RGC response to electrical stimulation was investigated using a post-stimulus time histogram (PSTH). RGC response strength was quantified by counting the number of electrically evoked RGC spikes per pulse, which is the difference between the number of spikes during the 400 ms before and after stimulation. The purpose of this study is to identify the abnormal network response to electrical stimulation in the degenerate retina, so we focused on the network-mediated RGC response by activation of the inner nuclear layer rather than the direct RGC response. To consider only network-mediated RGC responses, we disregarded directly evoked RGC spikes within 10 ms after electrical stimulation. The stimulation threshold was defined as the current amplitude when the number of evoked RGC spikes per pulse was 0.5. Threshold charge density was calculated using the following equation:


(2) $$\mathrm{Threshold}\,\mathrm{charge}\,\mathrm{density}=\frac{\text{I}\times \text{D}}{\pi \times r^{2}}$$

where *I* is the threshold amplitude, *D* is the pulse duration, and *r* is the MEA electrode radius (15 μm). The threshold charge and threshold charge density were always calculated from the 1st phase of the biphasic charge-balanced stimulus pulse. Electrical stimulation was applied to the retinal patches through one electrode of the MEA (mostly channel 44 in the middle of the MEA). The threshold charge density was measured for one stimulation electrode (Sekirnjak et al., 2006; Goo et al., 2011b; Abramian et al., 2014).

All results shown here, including spike frequency, inter-spike interval (ISI), LFP frequency, CCI, number of electrically evoked RGC spikes, threshold amplitude, threshold charge, and threshold charge density, were averaged for the analyzed neurons. Error bars indicate the mean ± standard error of the mean. A paired *t*-test was performed for statistical analysis between the two groups (*p < 0.05, **p < 0.01, and ***p < 0.001). Kruskal–Wallis test was performed with Tukey-Kramer, Bonferroni, and Scheffe *post hoc* tests (*p* < 0.05) to determine the statistical difference in CCI with inter-electrode distance and the number of evoked spikes with distance from the stimulation electrode.

## Results

### Spontaneous Activity of Retinal Ganglion Cells in Normal and Degenerate Retinas

To compare the spontaneous activity of RGCs in normal and degenerate retinas, we analyzed 572 RGCs from 14 retinal patches from wild-type mice and 555 RGCs from 13 retinal patches from *rd1* mice. For the different aging of *rd10* mice, we analyzed 288 RGCs from five retinal patches (P14 mice) and 252 RGCs from six retinal patches (P56 mice). Furthermore, to test whether the physiological properties of the degenerate retina observed in *rd* mice are conserved in non-human primate RD retinas, we compared the spontaneous activity between wild-type macaque retinas (231 RGCs, three retinal patches) and RD macaque retinas (179 RGCs, two retinal patches).

**FIGURE 1:**
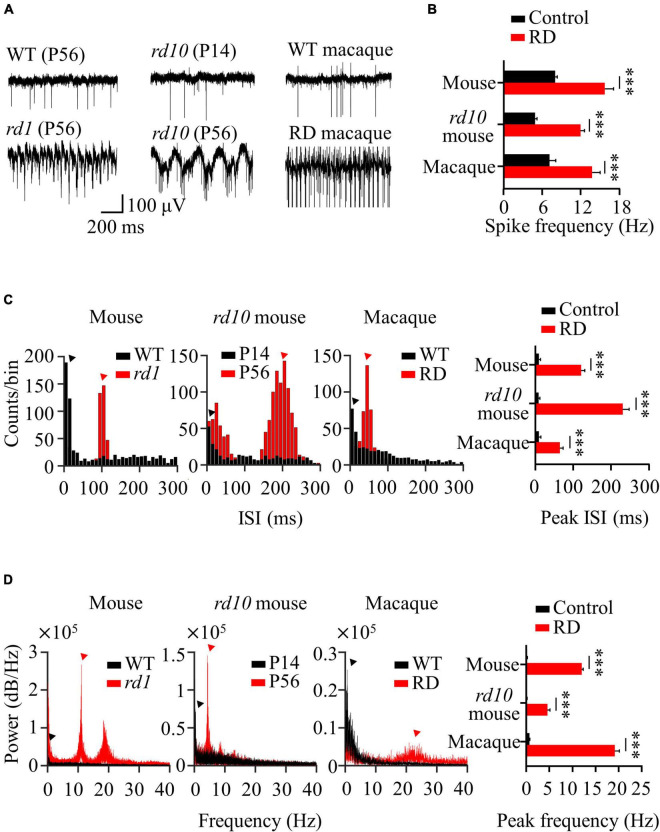
Spontaneous activity of RGCs in normal and degenerate retinas. **(A)** Representative raw traces of RGCs observed in control (WT P56, *rd10* P14, WT macaque) and RD groups (*rd1* P56, *rd10* P56, RD macaque). **(B)** Spike frequency of RGCs for control and RD groups. **(C)** Inter-spike interval histogram. Red and black arrowheads indicate the main ISI peaks for each RD and control group. **(D)** Power spectral density using FFT for detection of the dominant LFP frequency. Statistically significant differences between each of the different groups are shown above (***p < 0.001).

Degenerate RGCs showed hyperactive and rhythmic spontaneous activities compared to wild-type RGCs in both mouse and macaque retinas. First, degenerate RGCs showed hyperactive spontaneous firing compared with wild-type RGCs (Figures 1A,B). In the RD group, including *rd1*, *rd10* (P56), and RD macaque retinas, the spike frequency of degenerate RGCs was significantly higher than that of other controls (*p* < 0.001). Second, degenerate RGCs showed rhythmic bursts, each with different interburst intervals within the degenerate group (Figure 1A). Each degenerate group showed a distinct peak in ISI histogram compared with the wild-type group (*rd1*: 124.84 ± 9.86 ms, *rd10* P56: 235.45 ± 16.89 ms, RD macaque: 68.38 ± 8.76 ms) (Figure 1C). Third, degenerate RGCs showed abnormal oscillations in LFPs that were not found in wild-type RGCs (Figure 1A). The main peaks in the LFP power spectrum were observed at approximately 10 Hz in *rd1* (11.95 ± 0.36), 5 Hz in *rd10* P56 (4.54 ± 0.57), and 20 Hz in RD macaque (19.14 ± 0.99) (Figure 1D).

These results are in line with those of previous reports (Borowska et al., 2011; Trenholm and Awatramani, 2015; Goo et al., 2016). Several physiological characteristics such as hyperactivity, rhythmic bursts, and LFP oscillations in degenerate RGCs have been observed in *rd1* and *rd10* mice with the complete photoreceptor loss. However, in this study, new observations were made in RD macaque retinas, with complete disappearance of the outer segment and outer nuclear layer induced by MNU injection (OCT-based histological findings in Supplementary Figure 1). It would be interesting to discover these physiological properties in non-human primate models. This suggests that several physiological properties, including hyperactivity, rhythmic bursts, and LFP oscillations, are generally observed in degenerate retinas across species.

### Synchronized Firing Patterns Among Retinal Ganglion Cells in Degenerate Retinas

**FIGURE 2:**
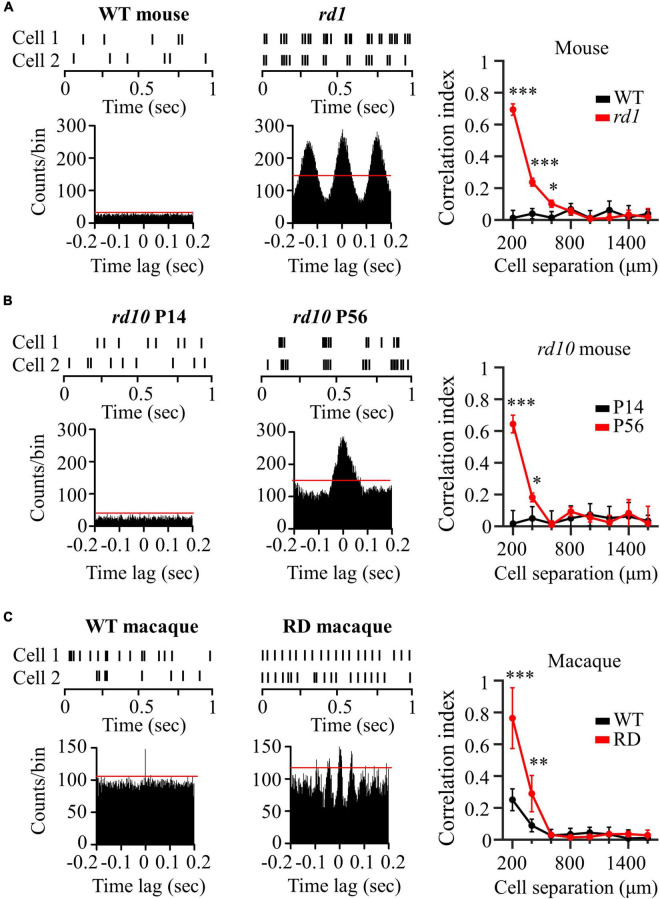
Degenerate RGCs showed more synchronized firing patterns. **(A)** Cross-correlation between two RGCs in wild-type and *rd1* retinas. Top left panel: raster plot of wild-type RGCs with 200 μm spacing on the MEA. Lower left panel: cross-correlogram between RGC pairs. The time bin of the histogram is chosen to be 2 ms. The red line indicates the significance level, indicated by the 99% confidence limit of the time histogram. Top middle panel: raster plot of *rd1* RGCs. Lower middle panel: cross-correlogram for *rd1*. Right panel: Cross-correlation index (CCI) as a function of inter-RGC distance. The time lag is chosen to be 0.2 s. Statistical differences between wild-type and *rd1* mice are indicated by asterisks (***p < 0.001, **p < 0.01, *p < 0.05). **(B)** Cross-correlation between two RGCs in *rd10* P14 and *rd10* P56. **(C)** Cross-correlation between two RGCs in wild-type and RD macaque.

We investigated synchronized firing between RGC pairs in the control and RD groups. Figure 2A shows representative spike trains of two RGCs spaced 200 μm apart in the wild-type and *rd1* mouse retinas. The *rd1* RGCs showed a strong spike correlation between the two RGCs, in contrast to wild-type RGCs. In the raster plot, the spikes in *rd1* RGCs were relatively well synchronized over time. This synchronized pattern is represented by significant peaks in the cross-correlogram. In contrast, wild-type RGCs showed no correlated firing between the two RGCs, even at the nearest spacing of 200 μm. In the cross-correlogram, wild-type RGCs did not show significant peaks, indicating few synchronized firing events.

To evaluate the strength of the spike correlations between the RGC pairs, we calculated the CCIs for each RGC pair recorded in the individual channels of the MEA. The right panel of Figure 2A shows CCI as a function of the inter-RGC distance. The CCI of *rd1* RGCs decreased with increasing distance between RGC pairs, whereas wild-type RGCs showed almost zero CCIs, regardless of the distance between RGCs. Significant differences in CCI between wild-type and *rd1* retinas were found for RGC pairs that were 200–600 μm apart. To determine the statistical difference in CCIs according to the inter-electrode distance, we performed Kruskal–Wallis test for wild-type and *rd1* retinas. Four statistically different groups (group 1, 200 μm; group 2, 400 μm; group 3, 600 μm; and group 4, 800–1,600 μm, *p* < 0.05) were found in the *rd1* retina, whereas no significant differences were found in the wild-type retina (*p* > 0.05).

Figure 2B shows the representative spike trains of the two RGCs in the *rd10* P14 and *rd10* P56 retinas. Similar to the results shown in Figure 2A, for P56 at the more progressive degeneration stage, RGC pairs showed strong synchronization compared to RGCs for P14 before retinal degeneration. Significant differences in CCI between *rd10* P14 and *rd10* P56 retinas were observed between RGC pairs spaced 200–400 μm apart. For Kruskal–Wallis test of *rd10* P56, three statistically different groups (group 1: 200 μm; group 2: 400 μm; and group 3: 600–1,600 μm, *p* < 0.05) were shown with inter-electrode distances.

In macaque monkeys, in contrast to wild-type mice and *rd10* P14 mice, wild-type macaque RGCs showed temporally narrow correlated firing of 2 ms in the cross-correlogram (left panel of Figure 2C). The CCI of wild-type macaque retinas showed statistical differences according to inter-electrode distance (group 1, 200 μm; group 2, 400 μm; group 3: 600–1,600 μm, *p* < 0.05) (right panel of Figure 2C). This finding is compatible with those of previous macaque and marmoset studies (Shlens et al., 2006; Ahn et al., 2020). Narrow correlations arise from direct connections through gap junction channels between RGCs (Brivanlou et al., 1998).

Interestingly, RD macaque RGCs showed a wider temporal (∼50 ms) correlation than did wild-type macaque RGCs (middle panel in Figure 2C). Previous studies have shown that broad correlations occur in indirect connections *via* the relays of inner retinal neurons, including bipolar or amacrine cells, which are upstream neurons of the RGC (Brivanlou et al., 1998). This suggests that connections between inner retinal neurons may be strengthened, indicating the potential for aberrant retinal remodeling in *rd* mice (Strettoi et al., 2003; Phillips et al., 2010; Choi et al., 2014). CCI in RD macaque retinas showed a statistically significant increase compared with wild-type macaque retinas for RGC pairs with 200–400 μm spacing (right panel in Figure 2C), consistent with those found in *rd* mice. Comprehensively, the degenerate retina has highly correlated RGCs compared to the wild-type retina in both mouse and macaque models.

### Oscillatory Rhythm Leads to Multiple Peaks of Electrically Evoked Retinal Ganglion Cell Responses in Degenerate Retina

**FIGURE 3:**
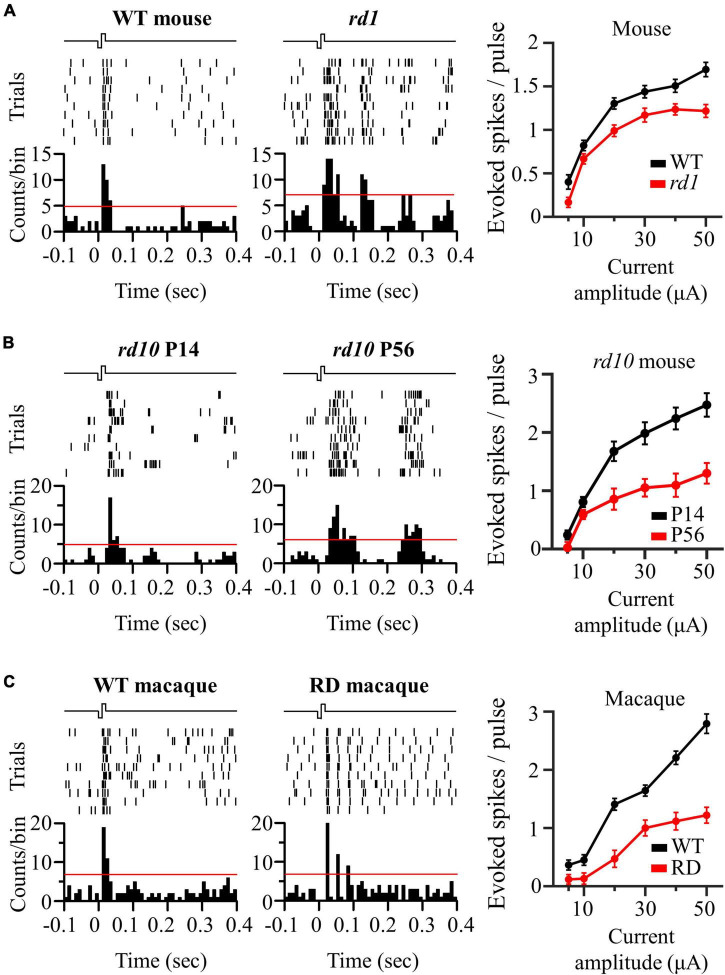
Oscillatory rhythm leads to multiple peaks of electrically evoked RGC responses in degenerate retina. **(A)** The responses of wild-type and *rd1* RGCs that are located 200 μm away from the stimulation electrode upon electrical stimulation with a pulse amplitude of 50 μA and a pulse duration of 500 μs. Top left panel: raster plot of wild-type RGC (10 trials). Lower left panel: post-stimulus time histogram (PSTH) accumulated over 10 trials. The time bin for PSTH is chosen to be 10 ms. The red line indicates the significance level, expressed as the 99% confidence limit for PSTH. Top middle panel: raster plot of *rd1* RGC. Lower middle panel: PSTH on *rd1*. Right panel: RGC response curve with increasing pulse amplitude. **(B)** The same representative figures with *rd10* P14 and *rd10* P56. **(C)** The same representative figures with wild-type and RD macaque.

We compared electrically evoked RGC responses between the control and RD groups. For the control group, the left panels of Figure 3 show representative RGC responses to electrical stimulation with a pulse amplitude of 50 μA and a pulse duration of 500 μs. Raster plots and PSTHs showed a single burst and one PSTH peak within 100 ms of the stimulation onset. In contrast, RD RGCs showed rhythmic bursts and multiple PSTH peaks at ∼10 Hz (*rd1*), ∼5 Hz (*rd10* P56), and ∼20 Hz (RD macaque) (middle panels in Figure 3), indicating that spontaneous oscillatory LFPs were also observed in the electrically evoked RGC responses.

The number of evoked RGC spikes was well modulated with increasing pulse amplitude in both the control and RD groups (right panels of Figure 3). At all pulse amplitudes, the number of evoked spikes was lower in the RD group than in the control group. Additionally, in terms of stimulation threshold parameters, including threshold amplitude, threshold charge, and threshold charge density, the RD group showed higher stimulation thresholds than did the control group (Table 1). Collectively, RD RGCs showed less response than control RGCs, suggesting that RD RGCs require higher stimulation charge injection to reach the levels of the wild-type RGC response in both mouse and macaque models.

**TABLE 1:** Comparison of threshold amplitude, threshold charge, and threshold charge density between control and RD groups.

	WT mouse	*rd1* mouse	*rd10* mouse (P14)	*rd10* mouse (P56)	WT macaque	RD macaque
Threshold amplitude (μA)	5.76 ± 0.64	10.47 ± 0.53 (***)	7.13 ± 0.45	9.68 ± 0.82 (**)	12.34 ± 0.91	25.75 ± 1.27 (***)
Threshold charge (nC/phase)	2.88 ± 0.32	5.24 ± 0.27 (***)	3.57 ± 0.23	4.84 ± 0.41 (**)	6.17 ± 0.46	12.88 ± 0.64 (***)
Threshold charge density (mC⋅cm^–2^/phase)	0.41 ± 0.05	0.74 ± 0.04 (***)	0.51 ± 0.03	0.68 ± 0.06 (**)	0.87 ± 0.07	1.82 ± 0.09 (***)

Statistical differences between control and RD groups are indicated by asterisks (***p < 0.001, **p < 0.01).

### Wide-Spreading Distribution of Electrically Evoked Retinal Ganglion Cell Population in Degenerate Retina

**FIGURE 4:**
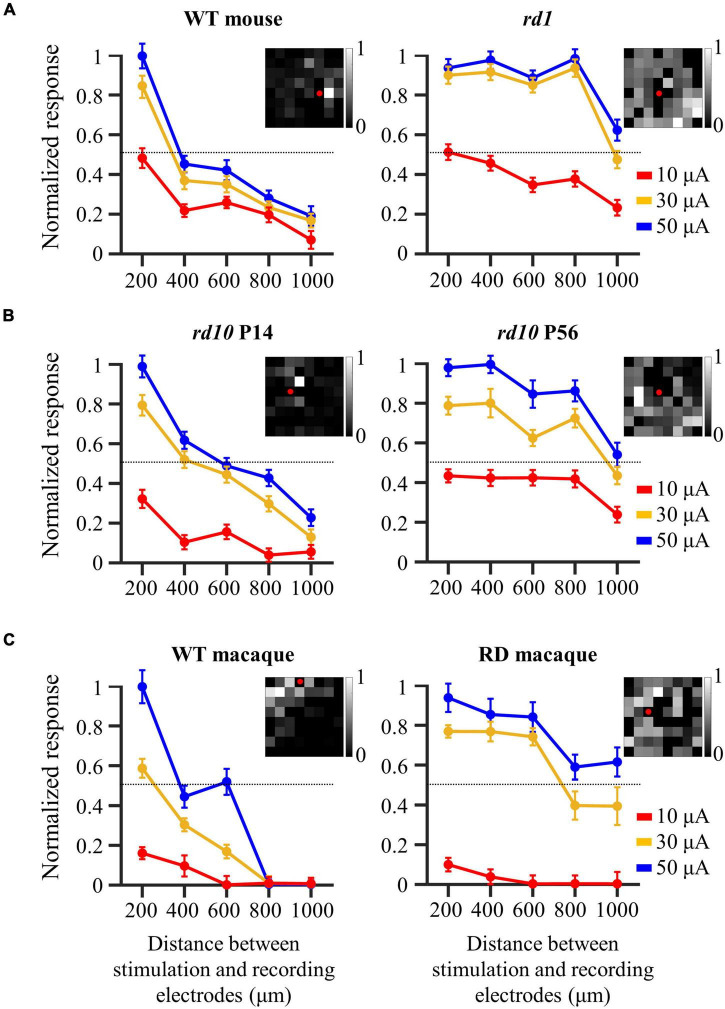
Wide-spreading distribution of electrically evoked RGC population in the degenerate retina. **(A)** Changes in normalized RGC response with the distance between stimulation and recording electrodes in wild-type and *rd1* mice. **(B)** Changes in normalized RGC response with the distance between stimulation and recording electrodes in *rd10* P14 and *rd10* P56. **(C)** Changes in normalized RGC response with the distance between stimulation and recording electrodes in wild-type and RD macaque. Insets in A, B, and C represent the spatial MEA colormap (grayscale) for normalized RGC responses upon electrical stimulation with a pulse amplitude of 50 μA and a pulse duration of 500 μs in one representative retinal patch. Red dots represent the position of stimulation electrodes.

The spatial distribution of the evoked RGC population was characterized by response intensity as a function of the distance between the stimulation and recording electrodes. The number of electrically evoked RGC spikes was normalized to the range [0 1] using feature scaling based on min–max normalization. Figure 4 compares the spatial changes in electrically evoked RGCs between the normal and degenerate retinas. Overall, RGCs in the control group showed a sharp decrease in response strength as they moved away from the stimulation electrode (left panels of Figure 4), suggesting that RGCs closer to the stimulation site were more effectively activated. Each spatial MEA color map for normalized RGC responses in one retinal patch showed a spatially confined RGC response to electrical stimulation (pulse amplitude: 50 μA, pulse duration: 500 μs). Contrastingly, RD RGCs showed a wide-spreading spatial distribution of electrically evoked RGC spikes, indicating a relatively gradual decreased pattern (right panels of Figure 4). Specifically, no change was noted in the response intensity of RD RGCs up to 800 μm (*rd1* and *rd10* P56) or 600 μm (RD macaques) to stimuli greater than 30 μA, except for a pulse amplitude of 10 μA. To quantify the spatial distribution of the RGC population in response to electrical stimulation, the distance from the normalized RGC response of 0.5 was used as the marginal distance for significant RGC activation. Normalized response stands for the normalized evoked spike number per pulse. Most RGCs in the control group had a distance of approximately 400 μm from the stimulation electrode (viewed at 30 μA, wild-type mice: 358 μm, *rd10* mice P14: 463 μm, wild-type macaque: 279 μm), whereas RD RGCs were more widely distributed (30 μA, *rd1* mice: 983 μm, *rd10* mice P56: 948 μm, RD macaque: 774 μm). Collectively, the normal retina showed a spatially confined RGC response to electrical stimulation, whereas the RD retina showed a spatially extended response to the same stimulus intensity.

### Origin of the Electrically Evoked Retinal Ganglion Cell Responses in the Degenerate Retina: Direct *vs.* Indirect

To investigate the origin of the electrical response shown in Figure 4, we performed a pharmaceutical experiment. Generally, there are two electrical responses. The first is the directly evoked RGC response. The other is a network-mediated response through a synaptic relay after photoreceptor or bipolar cell activation. However, only bipolar cell activation owing to complete photoreceptor loss at *rd1* P56 can be considered here. To evaluate whether the electrically evoked RGC response was direct or indirect, blockers of excitatory input to the RGCs (ionotropic glutamate receptor blockers: 50 μM DNQX and 50 μM DL-AP5) were applied to the perfusion solution.

**FIGURE 5:**
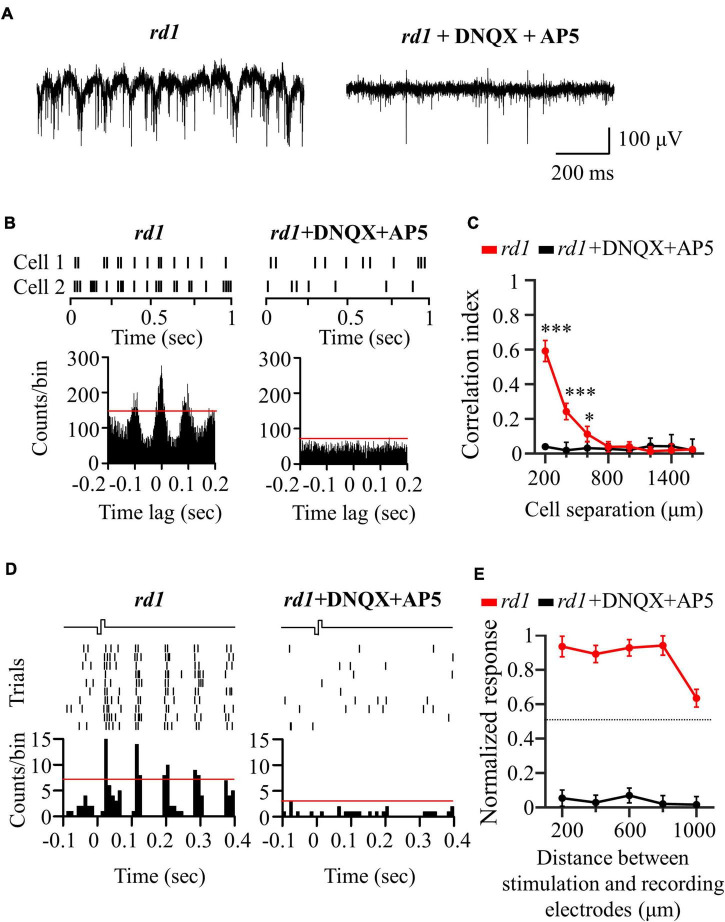
Application of glutamatergic receptor blocker (DNQX and DL-AP5) completely blocked network-mediated RGC responses in the *rd1* retina. **(A)** Representative raw traces of RGCs were shown for 1 s in *rd1* mice without and with glutamate receptor blockers. **(B)** Changes in synchronized firing between *rd1* RGCs after drug application. Top panels: raster plot of RGC with 200 μm spacing on the MEA. Lower panel: cross-correlogram between RGCs. The time bin for correlograms is chosen to be 2 ms. The red line indicates the significance level represented by the 99% confidence limit of the correlogram. **(C)** CCI as a function of inter-RGC distance. The statistical differences before and after drug application are indicated by asterisks (***p < 0.001, *p < 0.05). **(D)** The responses of RGC that is 200 μm away from the stimulation electrode to electrical stimulation with a pulse amplitude of 50 μA and a pulse duration of 500 μs. Top panels: raster plot of *rd1* RGC (10 trials). Lower panel: PSTHs. The time bin for PSTH is chosen to be 10 ms. The red line indicates the significance level, expressed as the 99% confidence limit for PSTH. **(E)** Normalized RGC response to electrical stimulation (pulse amplitude: 50 μA, pulse duration: 500 μs) with the distance between stimulation and recording electrodes without and with the presence of glutamate receptor blockers.

An additional six retinal patches and 343 RGCs were used in *rd1* mice for drug studies. First, Figure 5A shows the representative spontaneous activity of *rd1* RGCs recorded before and after the addition of blockers. The rhythmic bursts and LFPs disappeared within minutes after drug application, confirming their identity as a network origin. Figure 5B shows exemplary responses from the two RGCs spaced 200 μm apart. After drug application, synchronized firing between RGCs largely disappeared (Figure 5C). In addition, the electrically evoked RGC spikes completely disappeared when electrical stimulation was applied, suggesting that stimulation may involve presynaptic network activation in RGCs (Figures 5D,E). These findings indicate that the electrically evoked RGC spikes recorded in the degenerate retina are network-mediated and are not generated by direct RGC activation.

### Neuronal Mechanisms of Spatially Extended Retinal Ganglion Cell Responses in the Degenerate Retina

Our previous results showed that there were highly correlated RGCs in the degenerate retina (Figure 2). Furthermore, their synchronized firing and electrically evoked responses were network-mediated (Figure 5). Therefore, we hypothesized that the highly correlated retinal network caused spatial expansion of the RGC response to electrical stimulation, as shown in Figure 4. Previous studies on the degenerate retina have found that abnormal neural connections (retinal remodeling) occur specifically through the gap junction relay between ON-cone bipolar cells and AII amacrine cells (Menzler and Zeck, 2011; Margolis et al., 2014; Trenholm and Awatramani, 2015).

**FIGURE 6:**
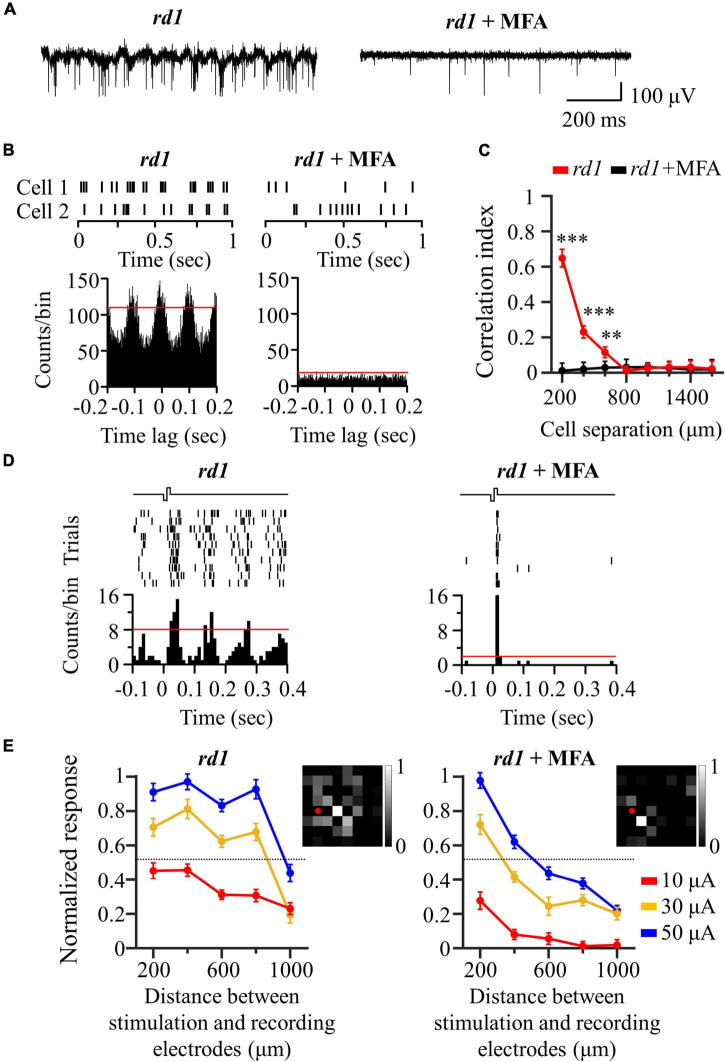
Application of gap junction blocker (MFA) blocked inner-retinal neuron coupling. **(A)** Representative raw traces of RGCs were shown for 1 s in *rd1* mice without and with gap junction channel blockers. **(B)** Changes in synchronized firing between *rd1* RGCs after drug application. Top panels: raster plot of two RGCs with 200 μm spacing on the MEA. Lower panels: cross-correlogram between RGCs. The time bin of correlograms is chosen to be 2 ms. The red line indicates the significance level represented by the 99% confidence limit of the correlogram. **(C)** CCI as a function of inter-RGC distance. Statistical differences before and after drug application are indicated by asterisks (***p < 0.001, **p < 0.01). **(D)** The responses of RGC that is 200 μm away from the stimulation electrode to electrical stimulation with a pulse amplitude of 50 μA and a pulse duration of 500 μs. Top panels: raster plot of *rd1* RGC (10 trials). Lower panel: PSTHs. The time bin for PSTH is chosen to be 10 ms. The red line indicates the significance level, expressed as the 99% confidence limit for PSTH. **(E)** Changes in normalized RGC response with the distance between stimulation and recording electrodes in the presence of gap junction channel blockers. Insets represent the spatial MEA colormaps (gray scale) for normalized RGC responses upon electrical stimulation with a pulse amplitude of 50 μA and a pulse duration of 500 μs in one representative retinal patch. Red dots indicate the position of stimulation electrodes.

To test our hypothesis, we applied MFA (100 μM) to block gap junction channels. An additional six retinal patches and 287 RGCs were used in the *rd1* mice. After MFA application, all rhythmic bursts, LFPs, and synchronized firing disappeared (Figures 6A–C). Moreover, multiple PSTH peaks observed during electrical stimulation disappeared, leaving only a single peak (Figure 6D). Concerning the spatial distribution of the electrically evoked RGC population, RGC activation became more localized after the application of gap junction blockers (Figure 6E), indicating that gap junctions indeed mediate spatially extended RGC responses in the degenerate retina.

## Discussion

**FIGURE 7:**
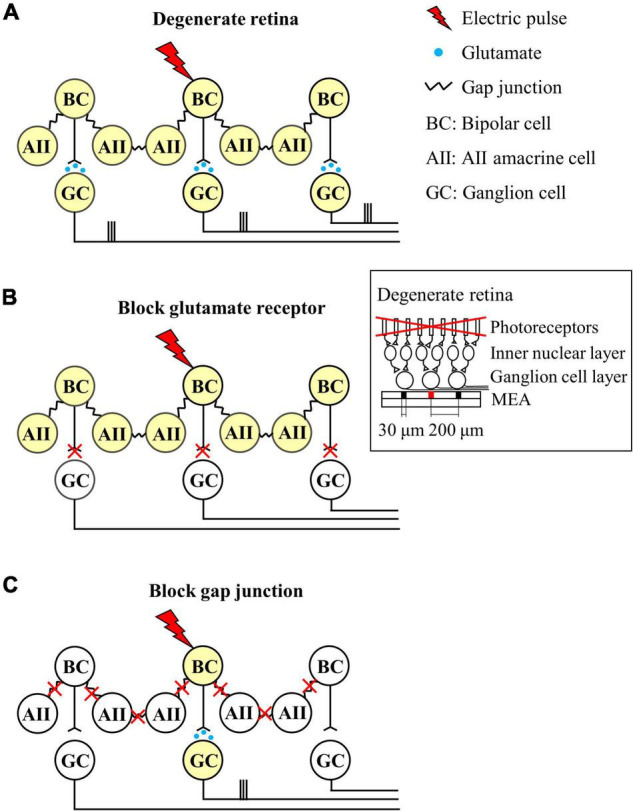
Schematic diagram of the neuronal mechanisms of spatially extended RGC responses in the degenerate retina. Different colors indicate whether the neuron is activated or not (white: no activation, yellow: activation). **(A)** Network response of the degenerate retina to electrical stimulation. Electrical stimulation activates the central bipolar cell, which excites synaptically connected RGC. The bipolar cell also activates AII amacrine cells through gap junction coupling, resulting in network activation. The network activation of the inner nuclear layer leads to widespread activation of remote RGCs. **(B)** Blockade of excitatory input to RGCs. **(C)** Decrease in gap junctional connectivity results in focal activation of bipolar cells and RGCs, but not extensive network activation. Therefore, RGC activity is localized in a confined area similar to the wild-type retina. Inset represents the schematic cross-section of the degenerate retina-MEA assembly. The retina is placed on top of the MEA with the RGC layer down. One central electrode (shown in red) is used for electrical stimulation, while all the others for recording electrically evoked RGC spikes. Electrode diameter = 30 μm, inter-electrode distances = 200 μm.

Figure 7 shows a schematic representation of our findings. When the RD retina is electrically stimulated, the activated bipolar cells transmit an evoked potential to the downstream RGCs in the vertical pathway, whereas the evoked potential of bipolar cells is also transmitted to gap junction-coupled inner retinal neurons (AII amacrine cells or bipolar cells). Accordingly, the gap junction-coupled retinal network activated the RGC population globally (Figure 7A). With the application of glutamatergic synaptic blockers (DNQX and DL-AP5), synaptic input from the inner retinal neurons to RGCs was blocked, and no network-mediated RGC responses were observed (Figure 7B). However, after the gap junction blockade (MFA), the evoked potential to the bipolar cell is transmitted only to the downstream RGC through a direct synaptic connection (Figure 7C).

In conclusion, our results show that degenerate RGCs become more correlated as retinal degeneration progresses. A more strongly coupled retinal network leads to a wider spatial distribution of the electrically evoked RGC spikes. Therefore, focal RGC activation is implausible in the degenerate retina, which is a major cause of low visual acuity in patients after electronic retinal implantation.

### Identification of Retinal Degeneration Stage Based on Abnormal Spontaneous Activity of Retinal Ganglion Cells

We found the abnormal spontaneous activity of RGCs in degenerate retinas from *rd1* P56, *rd10* P56, and RD macaque models (Figure 1). Degenerate RGCs showed higher spike firing rates and rhythmic bursts superimposed on oscillatory LFPs compared with controls, including wild-type mouse P56, *rd10* P14, and wild-type macaque models.

Our findings focus on the study of degenerate retinal networks in the intermediate RP stage. Many studies have noted abnormal spontaneous activity in the degenerate retina of *rd1* (Ye and Goo, 2007; Margolis et al., 2008; Stasheff, 2008; Menzler and Zeck, 2011; Trenholm et al., 2012; Yee et al., 2012) and *rd10* mice (Goo et al., 2011a; Stasheff et al., 2011; Biswas et al., 2014; Yoon et al., 2020). In particular, for *rd10* mice, the increase in spontaneous firing starts after ∼P20, when rod cells begin to die histologically, and remarkable hyperactivity was observed at ∼P45 when the rod cells almost disappeared (Gargini et al., 2007; Barhoum et al., 2008). However, a recent study following postnatal aging of *rd10* showed that spontaneous firing increased from P21 (Park et al., 2015), but there were no noticeable rhythmic bursts and oscillatory LFPs in most retinal patches until P30. A very small number of RGCs at P30 exhibited irregular LFP signals. LFP has been observed in *rd10* P45 (Goo et al., 2016). Therefore, we can infer rhythmic bursts and oscillatory LFPs as findings that indicate the onset of the intermediate RP phase when rod cells are almost lost. In this respect, our results focused on the intermediate RP stage (P56), when most photoreceptors have degenerated, but bipolar cells and RGCs remain intact.

Neural mechanisms underlying oscillatory LFPs have been studied in *rd1* and *rd10* mice. A well-known neural mechanism is that oscillations are an inherent physiological property of the electrically connected network between ON bipolar cells and AII amacrine cells (Borowska et al., 2011; Choi et al., 2014; Trenholm and Awatramani, 2015). This theory is understood by the fact that oscillatory LFP in the *rd* retina is inhibited by pharmacological blockade through the gap junction blocker, MFA. Thus, an electrically connected network between the ON bipolar cells and AII amacrine cells appears to be required to drive the LFP.

LFP was observed not only in transgenic *rd* mice but also in drug-induced rabbits (Ahn et al., 2019; Supplementary Figure 3) and macaque monkeys (Figure 1), in which rod cells were rapidly removed by MNU administration. The LFP was not observed in wild-type and drug-induced incomplete RD models (partial degeneration of the outer nuclear layer) but was found in the complete RD model (severe degeneration of the outer nuclear layer). Therefore, the LFP is not limited to a specific mouse model and can be a universal physiological marker for detecting changes in retinal networks that occur during the intermediate RP stage. However, this aberrant spontaneous RGC activity has not yet been studied *in vitro* and *in vivo* in human RP retinas. According to some studies, it has been reported that approximately 70% of patients with retinal degeneration see various visual symptoms, such as photopsia (brief flashes of light) and hallucinations of imaginary scenes (Heckenlively et al., 1988; Bittner et al., 2009; Brown et al., 2015). This abnormal visual perception may have been due to the appearance of spontaneously occurring RGC bursts superimposed on the LFP. Future studies in the retina of patients with RP are needed to elucidate the presence of abnormal spontaneous RGC activity, including hyperactive firing, rhythmic bursts, and LFP.

### Synchronized Firing Patterns Among Retinal Ganglion Cells in Normal and Degenerate Retinas

Retinal neurons collectively fire spikes in response to the visual stimuli. For example, during retinal circuits development prior to eye-opening, RGCs exhibit strongly synchronized spontaneous activity in the retina (Wong, 1999; Demas et al., 2003). Their synchronized activity refines immature visual circuitry and retinal projections to higher-order cortical neurons (Wong, 1999; Demas et al., 2003).

In the mature visual system, nearby RGCs show synchronous activity that depends on stimulus parameters including spot size, luminance, and contrast (Mastronarde, 1989; Neuenschwander et al., 1999; Hu et al., 2010). Synchronized firing patterns also differ between species (Pryluk et al., 2019; Ahn et al., 2020). For instance, marmoset monkey RGCs show strong correlations between RGC pairs, whereas mouse RGCs show no synchrony. In particular, Shlens et al. found that macaque RGCs showed a narrow correlation of spike firing with a time lag of 2 ms and that there was little broad correlation between RGCs (Shlens et al., 2006, 2009), consistent with our findings (left panel in Figure 2C). For spontaneous firing, it is understandable that each pair of RGCs in the macaque retina is directly connected *via* gap junction channels (Brivanlou et al., 1998).

In addition to normal neural networks, abnormal neural connections have also been observed in retinal degeneration. In the case of retinal degeneration due to the loss of photoreceptor cells, *rd1* and *rd10* mouse RGCs showed abnormally correlated activity (Menzler and Zeck, 2011; Margolis et al., 2014). Therefore, abnormal intrinsic correlations can often be utilized as physiological indicators of retinal degeneration.

Our results also showed that *rd1 and rd10* mouse RGCs exhibited strong synchronization compared to control RGCs (Figures 2A,B). Furthermore, RD RGCs showed highly correlated activity in macaque monkeys, a representative non-human primate model. Regarding why *rd1* retinas have significant correlations between RGCs up to 600 μm, whereas *rd10* P56 and RD macaques have correlations up to 400 μm (Figure 2), histologically, *rd1* almost loses its outer nuclear layer after P28 (Zhou et al., 2017). Conversely, *rd10* shows a remarkable decrease in rod cells at P45 (Gargini et al., 2007; Rosch et al., 2014); hence, *rd10* shows a slower degeneration than *rd1*. Therefore, it can be seen that, based on the same aging of P56, retinal degeneration and remodeling of *rd1* are more severe than that of *rd10*, and abnormal neural network formation progresses further. However, for the RD macaque retina, unlike the RD mouse retina with genetic modification, drug administration may not allow the retinal network of RD macaques to have sufficient time for rewiring. In this study, we used RD macaque retinas 12 weeks after MNU administration for acute photoreceptor degeneration. Given the difference in lifespan between mice and primates, the time required for retinal network changes owing to retinal degeneration is longer in primates than in mice (Jones et al., 2016). More than 12 weeks after MNU administration, it is expected that retinal network changes in RD macaques will progress further.

Regarding the limitations of our study regarding synchronized firing patterns, we did not observe significant differences in synchronized RGC firing in the control mouse group, even for the closest spacing of 200 μm. However, in previous studies, mouse RGCs have shown synchronized firing within ∼200 μm (Volgyi et al., 2013; Roy et al., 2017; Zhang et al., 2020). When using high-density MEA with an inter-electrode spacing shorter than 200 μm (Miccoli et al., 2019), we would expect to observe correlated firing between a pair of RGCs within a distance of 200 μm. Nevertheless, the fact that RD RGCs have highly correlated networks compared to normal RGCs is uncontroversial.

Furthermore, we could not subdivide RGCs into ON and OFF types since there is no light-evoked RGC response due to complete photoreceptor loss by retinal degeneration. As discussed above, our results focused on the intermediate RP stage, when most photoreceptors have degenerated, but bipolar cells and RGCs remain intact. No light responses were observed in RGCs, including *rd1* and *rd10* genetic mice at P56, and drug-induced macaque RD models. Further histological studies are needed to differentiate between ON and OFF types. Some studies have demonstrated successful cell-type classification into ON and OFF RGCs based on soma size and depth of dendritic stratification within the inner plexiform layer (Margolis et al., 2008, 2014). In future studies, more detailed cross-correlation studies of RGC types with histological findings should be performed.

### Spatially Extended Population Response to Electrical Stimulation in the Degenerate Retina

We observed a spatially extended response to electrical stimulation of the RD retina (Figure 4). As shown in Figures 5, 6, these RGC responses were transmitted through network activation, which was electrically connected to the inner retinal neurons. This phenomenon leads to the problem of unintentionally stimulating adjacent RGCs and consequently suppressing focal RGC activation.

Recently, simulation results were reported that compared the spatial resolution of reconstructed images between two groups by decoding electrically evoked RGC responses of healthy and RD retinas. The simulation showed that healthy retinas had good spatial resolution when reconstructing the RGC population response into visual scenes, whereas RD retinas had low spatial resolution (Golden et al., 2019). This is the first study to use computational modeling to explain the phenomenon of decreased visual acuity in patients with RP during electrical stimulation. In line with this, our results provide experimental evidence that explains the low spatial resolution of retinal coding for electrical stimulation. This RGC population response is transmitted to the visual cortex, a higher-order cortical nucleus, resulting in spatially low-resolution vision.

Relatively low stimulus intensity is required to avoid excessive activation of the retinal network. Based on the results of Figures 4, 6 and Ryu et al. (2017), when a current of ∼10 μA was stimulated to the retina, it was able to induce a relatively spatially localized RGC population response (Ryu et al., 2017). However, the problem is that stimulation of the retina with a current intensity that is too low cannot guarantee a stable RGC response owing to the low SNR. This can be confirmed by the fact that the number of spikes evoked for a 10 μA stimulus was less than 1 (Figure 3).

Of course, the stimulation thresholds obtained in this study may be rather high, since the stimulation thresholds were derived from RGC responses that were at least 200 μm apart, in that stimulation and recording cannot be performed simultaneously from one electrode due to saturation of stimulation artifacts. Nevertheless, given the stimulus charge levels used in clinical trials for patients with RP (above the maximum charge of 25 nC used in this study) (Humayun et al., 2012; Chow, 2013; Fujikado et al., 2016), spatially extended population responses are expected to occur in the RD retina. This results in low visual acuity and low spatial resolution.

Furthermore, the stimulation pulse rate of 1 Hz used in this study is lower than clinically used pulse rates (at least 5 Hz or higher). Depending on which retinal neuron is stimulated, approximately 5 Hz is used for sub-retinal prosthesis, which stimulates the inner nuclear layer to induce network-mediated RGC responses (Zrenner et al., 2011; Chuang et al., 2014; Stingl et al., 2015).

The purpose of this study is to find the abnormal network response (wide-spreading spatial distribution of electrically-evoked RGC spikes) observed in the degenerate retina during electrical stimulation and to elucidate the neural mechanism of this phenomenon. Therefore, in this study, we tried to minimize the desensitization effect by choosing 1 Hz stimulation. In our previous publication, we applied electrical stimulation to *rd1* mouse retinas at P56 while increasing the pulse rate from 1 Hz to 10 Hz (Ryu et al., 2009). We observed a rapid decrease in electrically-evoked RGC spikes at a pulse rate of 5 Hz or higher even if the same pulse duration stimulation was applied to the degenerate retina. This desensitization of RGC responses to high-frequency stimuli has also been confirmed in other studies (Freeman and Fried, 2011; Im and Fried, 2016; Chenais et al., 2021a). Specifically, Chenais et al. (2021a) found that when the same pulse duration stimulation at high frequencies (1–20 Hz) was continuously applied to the *in-vitro rd10* mouse retinas at ∼P120, the network-mediated RGC responses became insensitive to stimuli above 5 Hz. Specifically, the number of electrically-evoked RGC spikes during high-frequency stimulation decreased rapidly after the first stimulation (Figure 3 of Chenais et al., 2021a). In Chenais et al’s study, a strategy to suppress RGC desensitization using time-varying, non-stationary stimulation (pulse duration modulation) was proposed.

Since the RD group showed spatially expanded responses of the RGC population compared with the control group at the same 1 Hz stimulation, there is a high possibility that the abnormal network responses are still maintained with higher pulse rates (>1 Hz). In future studies, we would like to observe if the spatially expanded RGC response still appears in the degenerate retina when applying a clinically used pulse rate with time-varying, non-stationary stimuli proposed by Chenais et al. (2021a). Whether the observed wide-spreading spatial distribution of electrically-evoked RGC spikes in RD retina is maintained, enhanced, or suppressed during high frequency stimulation will be of great interest in the artificial vision community.

### Clinical Implication for Retinal Prosthesis

The electrode location on the retinal layer determines one of two widely used stimulus configurations. One is epi-retinal and the other is a sub-retinal stimulus configuration. Epi-retinal stimulation directly activates RGCs, whereas sub-retinal stimulation primarily targets the surviving photoreceptors or intact bipolar cells. Epi-retinal stimulation has the advantage of enabling relatively simple RGC activation compared to complex network activation. However, the disadvantages are that RGCs fire only one spike regardless of stimulation intensity (non-naturalistic response) and non-selective activation of RGC axon bundles passing between the RGC layer and the stimulation electrode (Fried et al., 2009; Beyeler et al., 2019). In contrast, sub-retinal stimulation has the advantage of being suitable for mimicking natural retinal responses through network activation (Im and Fried, 2015). However, if retinal degeneration is severe and the inner nuclear layer containing bipolar cells is not preserved, sub-retinal stimulation is impractical.

Directly targeting RGCs can avoid problems associated with spatially extended RGC responses based on abnormal network activation. An *in vitro* study of *rd10* P130-260, mimicking the late RP stage, showed a spatially restricted RGC response through direct RGC activation (Corna et al., 2021). On the contrary, network activation targeting the inner nuclear layer appears to be problematic because of the highly correlated retinal networks after retinal degeneration has progressed severely. To avoid these issues, prosthetic implantation is recommended for patients in the early RP stage. Artificial retinal devices have been used in early-stage patients whose photoreceptors are not fully degenerated. Even in *in vitro* experiments, the activated RGC population showed spatially confined responses from *rd1* to P20 (Haq et al., 2018).

Nevertheless, solutions that suppress highly correlated RD networks should be considered if the scope of prosthetic coverage is not limited to the initial stage and needs to be expanded. The first step was to eliminate rhythmic bursts and local field potentials observed in the RD retina. Some studies have shown that the SNR of a single RGC response upon electrical stimulation improves after the application of drugs, such as MFA and benzodiazepines, with the disappearance of abnormal spontaneous activity (Toychiev et al., 2013; Eleftheriou et al., 2017; Gehlen et al., 2020). Our results, considering the response of the RGC population to electrical stimulation, demonstrated the effectiveness of drug treatment with MFA (Figure 6). However, in some clinical trials, drug treatment may be limited owing to unknown side effects, such as cytotoxicity.

Therefore, there is a need for a new electrical stimulation strategy that eliminates abnormal spontaneous activity with only an implanted electronic chip without the aid of drugs. It has been reported that high-amplitude electrical stimulation or asymmetric pulse stimulation targeting ON-bipolar cells (based on a simulation study) can suppress the aberrant spontaneous activity of the RD retina at the single-cell level (Haselier et al., 2017; Loizos et al., 2018). Conversely, abnormal neural activity, including local field potential and synchronization between neurons, is commonly found in neurological diseases such as Parkinson’s disease and epilepsy in addition to retinal degeneration (Lian et al., 2003; Rubchinsky et al., 2012; Wang et al., 2016). For therapeutic approaches, various types of electrical stimulation have been attempted, such as closed-loop systems for antiphase LFPs (Sanabria et al., 2020) and high-frequency stimulation above 100 Hz (Santaniello et al., 2015; Ma et al., 2019), to suppress aberrant activity in these neuropathological fields. As a breakthrough to overcome low-resolution vision in patients with RP, it may be promising to apply therapies developed in other neurological fields to retinal degeneration. Our results may serve as an initial database for further therapeutic studies.

## Conclusion

We studied the aberrant network-mediated responses of RGCs to electrical stimulation using multielectrode arrays. First, no correlation was observed between RGCs in the control group, including wild-type mice, *rd10* P14 mice, and wild-type macaque retinas. Contrastingly, in the RD group, including *rd1, rd10* P56, and RD macaque retinas, RGCs were significantly correlated. Second, the number of electrically evoked RGC spikes decreased exponentially in the control group with the distance between the stimulation and recording electrodes, whereas electrically evoked RGC spikes were observed throughout the retina in the RD group regardless of the inter-electrode distance. Our results showed that degenerate RGCs were more correlated as retinal degeneration progressed. Highly correlated retinal networks lead to spatial expansion of RGC responses to electrical stimulation. It interferes with focal activation of RGCs in the degenerate retina, resulting in low-resolution vision in RP patients with retinal prostheses. This should be considered in the future to improve the visual acuity of prosthesis-implanted patients.

## Data Availability Statement

The raw data supporting the conclusions of this article will be made available by the authors, without undue reservation.

## Ethics Statement

The animal study was reviewed and approved by Institutional Animal Care Committee of Chungbuk National University and Institutional Animal Care Committee of the OSONG KBIO HEALTH.

## Author Contributions

JA, YY, and YSG conceived the study. JA, SC, K-EC, and S-WK conducted the experiments. JA, SC, and K-EC analyzed the data. JA, S-WK, YY, and YSG prepared the manuscript. All authors contributed to the article and approved the submitted version.

## Conflict of Interest

The authors declare that the research was conducted in the absence of any commercial or financial relationships that could be construed as a potential conflict of interest.

## Publisher’s Note

All claims expressed in this article are solely those of the authors and do not necessarily represent those of their affiliated organizations, or those of the publisher, the editors and the reviewers. Any product that may be evaluated in this article, or claim that may be made by its manufacturer, is not guaranteed or endorsed by the publisher.
