# Supplementary material for: Correlated Activity in the Degenerate Retina Inhibits Focal Response to Electrical Stimulation
Source: Front Cell Neurosci. 2022 May 4;16:889663. doi: 10.3389/fncel.2022.889663 (PMC9114441; doi:10.3389/fncel.2022.889663)
Supplement: Supplementary file 1 [file Data_Sheet_1.DOCX]

Supplementary Material

**
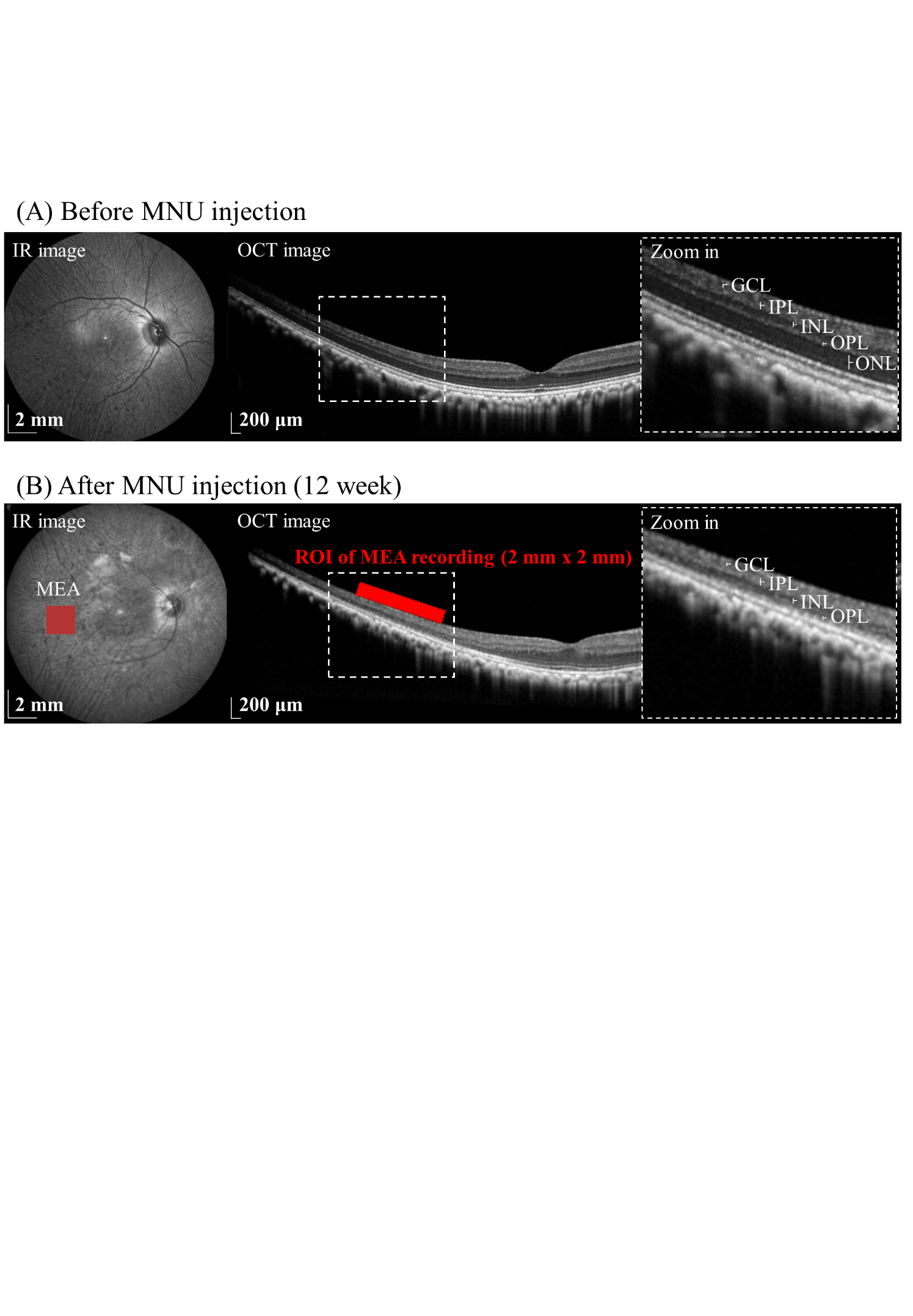
**

**Supplementary Figure 1.** Retinal anatomical changes in a representative case of MNU-induced macaque RD model using OCT imaging. **(A)** Infrared images and OCT images before MNU injection are shown. The enlarged image with a white dashed line for the peripheral region is shown as each sublayer. All sublayers including GCL, IPL, INL, OPL, and ONL are well visible. **(B)** Infrared images and OCT images are shown 12 weeks after surgery. The inner retinal layer including GCL, IPL, INL are well observed in the enlarged image of the periphery. Weak OPL is observed. In contrast, the ONL in the periphery is severely damaged. For *in-vitro* MEA recording, a retinal patch was isolated from the periphery (red square). For OCT image, the region of interest (ROI) of MEA recording is indicated by a red rectangle. GCL: ganglion cell layer; IPL: inner plexiform layer; INL: inner nuclear layer; OPL: outer plexiform layer; ONL: outer nuclear layer.

**
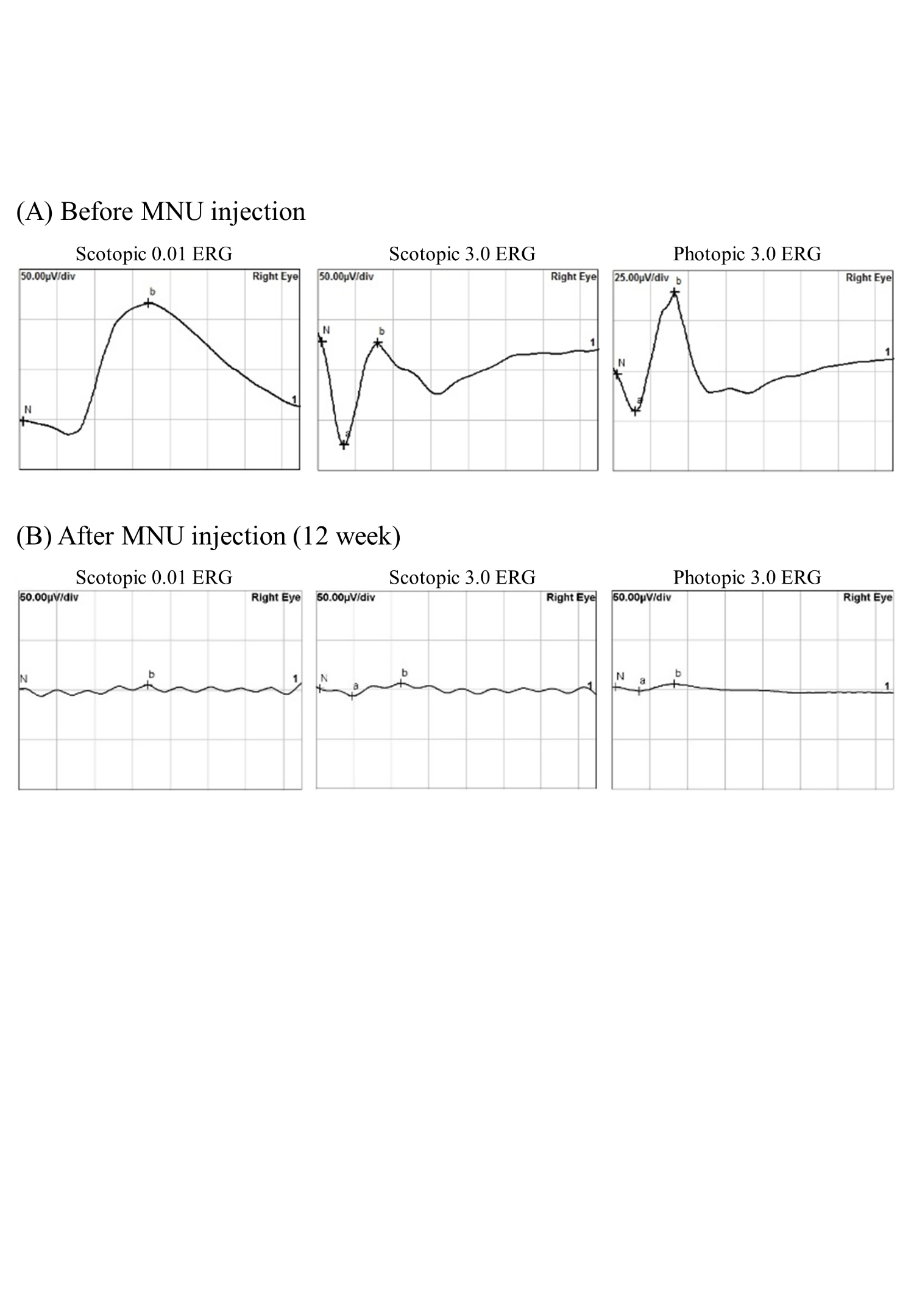
**

**Supplementary Figure 2.** ERG changes in a representative case of MNU-induced macaque RD model. The first, second, and third columns represent scotopic 0.01 ERG, scotopic 3.0 ERG, and photopic 3.0 ERG, respectively. **(A)** ERG graph before MNU injection is shown. The peak latency and amplitude of the b-wave at the scotopic 0.01 ERG are 69 ms and 117 μV. For scotopic 3.0 ERG, a-wave (14.1 ms, 103 μV) and b-wave (32.0 ms, 101 μV). For photopic 3.0 ERG, a-wave (12 ms, 18.1 μV) and b-wave (32.9 ms, 58.9 μV). **(B)** ERG graph 12 weeks after surgery are shown. Most scotopic ERG graphs show severely reduced amplitude. For scotopic 0.01 ERG, b-wave (68.4 ms, 4.45 μV). For scotopic 3.0 ERG, a-wave (19.4 ms, 7.50 μV) and b-wave (45.5 ms, 12.9 μV). For photopic 3.0 ERG, a-wave (14.7 ms, 4.32 μV) and b-wave (33.5 ms, 7.36 μV).


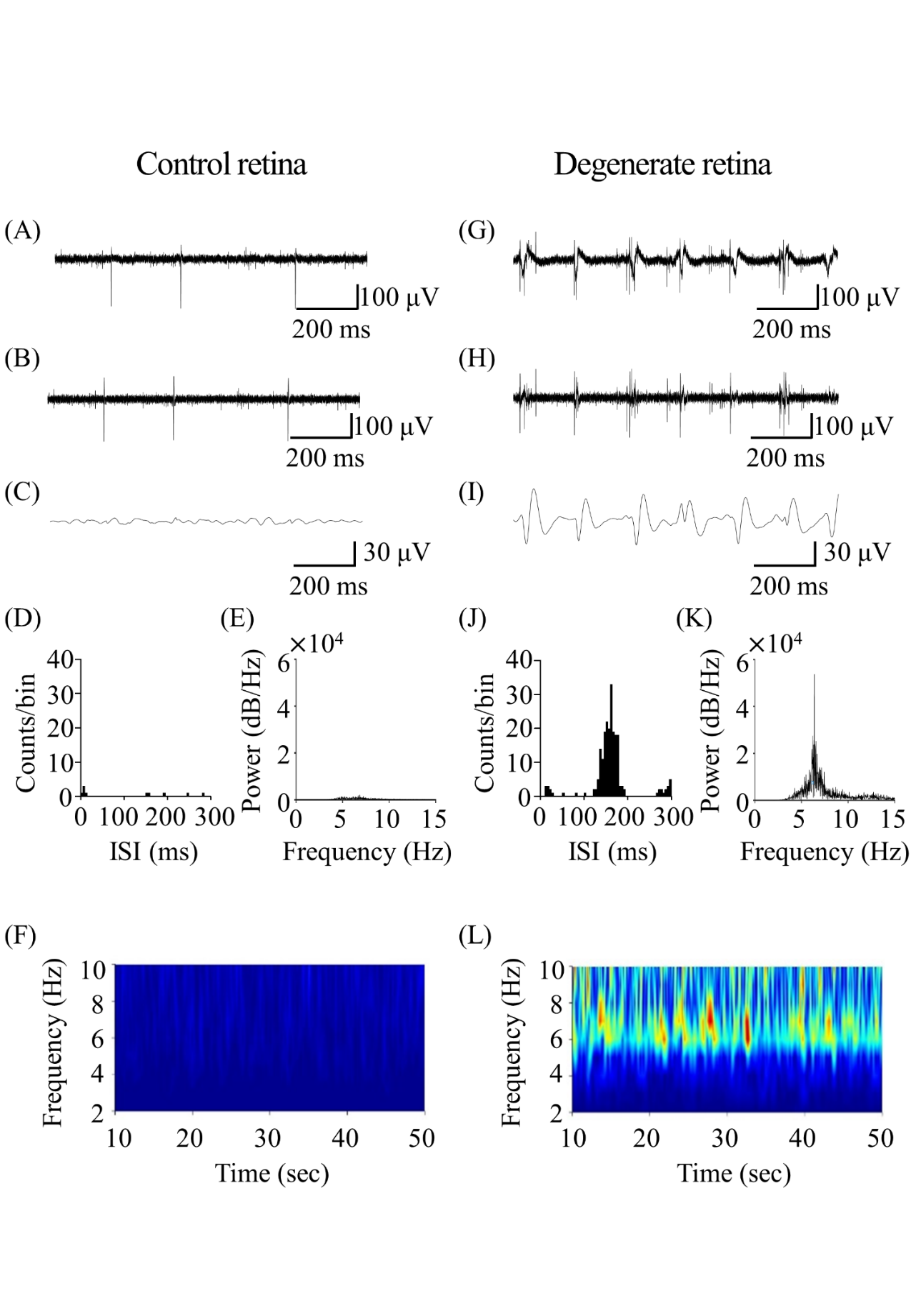


**Supplementary Figure 3.** Spontaneous activities in MNU-induced non- and degenerate retinas in the right eye of rabbits. New Zealand white rabbits received an intravitreal injection of MNU (0.2 mg in 0.05 mL of phosphate-buffered saline) two weeks after vitrectomy in the right eye. **(A)** Representative raw traces of RGCs observed for 1 sec in a control retina. **(B)** RGC spiking obtained by high-pass filtering with a 100-Hz cutoff frequency. **(C)** Local field potential (LFP) traces obtained from low-pass filtering with a 40-Hz cutoff frequency. **(D)** Inter-spike interval histogram. **(E)** Power spectral density using FFT for LFP frequency detection. **(F)** Power spectrum through continuous wavelet transform over period. A hotspot represents a dominant oscillatory frequency. **(G-L)** Degenerate retina. Degenerate RGCs showed hyperactive spontaneous firing compared with control retina, and rhythmic bursts superimposed on oscillatory LFPs were also observed. The dominant peak of ISIH was around 150 ms, which corresponds to ~ 6.6 Hz in the frequency domain.
